# Seroprevalence and risk factors associated with *Leishmania infantum* in dogs in Sardinia (Italy), an endemic island for leishmaniasis

**DOI:** 10.1007/s00436-020-06973-0

**Published:** 2020-11-17

**Authors:** Claudia Tamponi, Fabio Scarpa, Silvia Carta, Stephane Knoll, Daria Sanna, Carolina Gai, Anna Paola Pipia, Giorgia Dessì, Marco Casu, Antonio Varcasia, Antonio Scala

**Affiliations:** 1 Parassitologia, Dipartimento di Medicina Veterinaria, Università degli Studi di Sassari, Via Vienna, 2, 07100 Sassari, Italy; 2 Dipartimento di Scienze Biomediche, Università degli Studi di Sassari, Sassari, Italy

**Keywords:** Leishmaniasis, Endemic areas surveillance, Sardinia, dogs

## Abstract

Leishmaniasis is a widespread, vector-borne parasitosis causing clinical manifestations in animals and in humans. In dogs, Canine Leishmaniasis has been reported in as much as 50 countries and the Mediterranean basin is known to be one of the most affected zones. Within these areas, the Island of Sardinia (Italy) has long been considered endemic for leishmaniasis and the presence of two arthropod vectors has recently been reported there. Nevertheless, to date, no epidemiological surveys regarding CanL have been carried out on the island. Hence, for the first time, the seroprevalence and the risk factors were investigated. Blood samples, as well as clinical and general information from 1.147 dogs, were collected and analyzed. Dogs consisted of two distinct populations, namely “owned dogs” and “kennel dogs.” Anti-*Leishmania* IgG antibodies were detected using IFAT and samples were scored as positive at a cut-off dilution of 1:80. Data was analyzed using a Chi-squared test and bivariate and multivariate analyses were performed. Overall, 15.4% of dogs were found to be infected with CanL while only 44.1% of these animals exhibited clinical signs. Owned dogs (27.2%) were found to be infected more often than kennel dogs (10.6%); male dogs were found to be more frequently infected than female dogs and the number of infected animals increases with age. The present survey confirmed the endemic nature of leishmaniasis in Sardinia with a similar seroprevalence as mainland Italy. The results obtained serve as validation for the hypothesis that, in endemic areas, clinical CanL representations constitute only a fraction of the leishmaniasis cases.

## Introduction

Leishmaniasis are a group of vector-borne diseases caused by *Leishmania* spp., commonly affecting several animal species and humans in more than 88 countries (Khan et al. 2020). On a global scale, 0.35 billion individuals are continuously at risk of acquiring the disease (mostly cutaneous and mucocutaneous leishmaniasis) with an expected predominance of 12 million cases and a yearly incidence of 1.5 million cases (Khan et al. 2020; Alvar et al. 2012).

Thought the distribution of *Leishmania* spp. consists typically of tropical and subtropical regions, these parasites are expanding to new areas, e.g., within Central Europe and the Americas (Dantas-Torres et al. 2012; Ferroglio et al. 2018).

In the Mediterranean basin, two zoonotic species, namely *Leishmania infantum* and *Leishmania major*, and two anthroponotic species, *Leishmania donovani* and *Leishmania tropica*, are present (Maroli et al. 2013).

Canine Leishmaniasis (CanL) has been reported in 50 of the 88 countries in which human leishmaniasis is endemic and the Mediterranean basin is one of the most affected areas among them (Alvar et al. 2004; Morales-Yuste et al. 2012).

At least 2.5 million dogs are infected with *L. infantum* in southwestern Europe (Moreno and Alvar 2002; Baneth et al. 2008; Pennisi 2015) as well as various species of sylvatic hosts (Molina et al. 2012; Ferroglio et al. 2018).

Within the Mediterranean basin, on the island of Sardinia (Italy), an endemic region for leishmaniasis (Gramiccia 2011), over 250 human cases were reported between 1922 and 2014 (Maroli et al. 1994; Ferreli et al. 2004; Madeddu et al. 2014). More recently, an atypical leishmaniasis case involving a 57-year-old woman with Down’s syndrome (Ferreli et al. 2004) and a case of mucocutaneous leishmaniasis as part of the presentation of an HIV infection were described (Madeddu et al. 2014). Phlebotomine sand flies (Diptera: Psychodidae) are the only arthropods adapted for the biological transmission of *Leishmania* (Desjeux 1996), and 42 species regarded as either proven or putative vectors of these parasites have been identified (Maroli et al. 2013). Within the endemic areas of Europe, CanL is erratically distributed with a high variability of infection prevalence between hypoendemic and hyperendemic foci. In Italy, classical endemic zones of CanL include southern and central regions of the country, together with the islands of Sicily and Sardinia (Gramiccia 2011). However, for the last decades, CanL has been characterized by a Northward spread toward areas previously considered as nonendemic (Maroli et al. 2008; Santi et al. 2014), reaching as far as the foothills of the Alps in northern Italy and the Pyrenees in southern France and northern Spain (Solano-Gallego et al. 2011). This expansion is due to the ability of *Leishmania* spp. to spread rapidly and extensively within exposed dog populations as long as environmental conditions allow for vector activity (Baneth et al. 2008; Dantas-Torres et al. 2012). Furthermore, the large numbers of dogs traveling to southern Europe, as well as those imported as companion animals from areas where CanL is endemic, have contributed to the increasing number of clinical cases in nonendemic countries such as the United Kingdom and Germany (Solano-Gallego et al. 2011).

Even though several regional reports have described CanL as endemic to Sardinia (Maroli et al. 1994) and the presence of two proven *L. infantum* vectors (i.e., *Phlebotomus perfiliewi* and *P. perniciosus*) has recently been reported in urban, peri-urban, and rural environments on the island (Carta et al. 2020), no epidemiological surveys on CanL have been carried out on Sardinia to date, while two papers on leishmaniasis in cats were recently published (Ennas et al. 2012; Dedola et al. 2018). Furthermore, besides the obvious risks to the human and animal population on the island, updating the epidemiological knowledge concerning CanL on Sardinia is of paramount importance to, due to the touristic appeal of this region, which attracts millions of travelers (and their pets) each year, including many coming from non-endemic regions (SIRED 2019).

Therefore, the aim of the present paper is to fill in the gaps in the current scientific knowledge regarding CanL on the Mediterranean island of Sardinia by assessing the seroprevalence and the risk factors associated with CanL among kennel and owned dogs of the region.

## Material and methods

### Study area and dog population

The present study was carried out on the island of Sardinia, Italy, and data for this research were collected between 2012 and 2018. The study area included the whole territory of Sardinia, involving the provinces of Sassari, Nuoro, Oristano, and Cagliari. Sardinia is the second largest island in the Mediterranean Sea and its climate is characterized by hot and dry summers with mild and wet winters (Köberl et al. 2016). Current population statistics ascertain a total human population of 1,639,591 individuals (Sardegna Statistiche 2019) and a canine population of 542,224 (http://www.salute.gov.it). During this research, solely dogs permanently residing within the borders of Sardinia and older than six months of age were sampled. Animals included in this survey were divided into the following two groups; (i) dogs residing within kennels (from here referred to as “kennel dogs”) and (ii) dogs which were referred to the Veterinary Teaching Hospital of the University of Sassari for clinical or routine veterinary analysis (from here referred to as “owned dogs”). Seven different animal shelters in multiple localities on Sardinia (Santa Maria la Palma, Porto Torres, Codrongianos, Sassari, Cagliari, Olbia, and Narbolia) were sampled. Formal permission for the collection and use of samples within the context of this research was obtained from the owners of the animals when relevant.

### Sampling and serological study

Each dog included in this research underwent a complete clinical examination performed by a veterinarian with a focus on clinical signs indicative of CanL. Animals were classified as dogs with clinical signs if general clinical signs (poor body condition, lymphadenomegaly), cutaneous and mucocutaneous lesions, truffle hyperkeratosis, onychogryphosis, and/or ocular lesions were found.

Additional data (sex, age, lifestyle, body size, and length of hair) on each animal was collected, as well as information on habitat, outside access, and the presence or absence of nocturnal refuges. Habitat was defined as either urban or rural and the presence/absence of nocturnal refuges as dogs spending the night indoors or outdoors respectively. Furthermore, owners were questioned regarding the use of prophylactic measures against sand flies, like topical repellents.

Peripheral blood samples (total of 5 ml) for this research were collected by cephalic venepuncture. Refrigerated samples were transported to the laboratory and serum was obtained from each sample by centrifugation before subsequently being stored at − 20 °C awaiting antibody testing.

Anti-*Leishmania* IgG antibodies were detected using an in-house Immunofluorescent Antibody Test (IFAT) according to the laboratory procedures described in the OIE Manual of diagnostic Tests and Vaccines for Terrestrial animals (OIE 2018). Promastigotes of *L. infantum* zymodeme MON-1 were used as antigen and the dilution started from 1:40. The serum from a sick dog with a confirmed infection was included as positive control.

Samples were scored as positive for CanL when these produced a clear cytoplasmic and membrane fluorescence for promastigotes from a cut-off dilution of 1:80, according to Italian National Reference Centre for Leishmaniasis (C.Re.Na.L. – Istituto Zooprofilattico Palermo, Italy) as described by Foglia Manzillo et al. (2018). Positive sera were titrated until negative results were obtained. The highest dilution showing fluorescent promastigotes was taken to be the antibody titer, whereas samples solely showing fluorescence at 1:40 dilution were considered exposed but not infected. All data collected was finally compared to published CanL infection rates. All testing was conducted within the veterinary parasitology laboratory of the Department of Veterinary Medicine at the University of Sassari.

### Statistical analysis

Differences in the prevalence of seropositive dogs related to sex, age, breed, size, and dog’s hair length were compared by using Pearson's Chi-squared test (with Yates’ continuity correction) on the whole dataset including both owned and kennel dogs. Differences were considered statistically significant for *P* < 0.05.

In order to test the relationships between infection and the available variables, bivariate and multivariate analyses were performed on two datasets independently: (i) owned dogs and (ii) kennel dogs.

Data related to owned dogs were analyzed by means of nine variables: sex (male/female), age (< 1 to 20 years), breed (purebred/crossbred), size (small/medium/big), dog’s hair length (long-haired/short-haired), prevention (yes/no), type of habitat (rural/urban), day shelter (indoors/outdoors), and night shelter (indoors/outdoors). Data related to kennel dogs were analyzed by means of five variables: sex (male/female), age (< 1 to 20 years), breed (purebred/crossbred), size (small/medium/big), and dog’s hair length (long-haired/short-haired).

The multivariate regression analysis was performed applying a backward selection procedure in which non-significant covariates were deleted step by step, until to the final model was attained, which was used to estimate the odds ratio with 97.5% C.I. (confidence interval). Both bivariate and multivariate approaches were applied using the regression analysis performed by means of a Generalized Linear Mixed Models (GLMM) implemented in the R-package lme4 (Bates et al. 2015) in R environment version 3.6.3 (R Core Team available at https://www.r-project.org/). Statistical significance was set at *P* < 0.05. GLMMs was also used to reach a final model capable of detecting useful covariate predictor for the estimation of infection odds in exposed dogs. Finally, Pearson (*r*) correlations were used to assess the relationship between the occurrence of clinical signs and infection.

## Results

A total of 1147 dogs were sampled during this research, including 812 dogs from kennels (Santa Maria la Palma = 370; Porto Torres = 155; Codrongianos = 124; Sassari = 65; Cagliari = 45; Olbia = 28; Narbolia = 25) and 335 owned dogs. The total sampled population was balanced in terms of sex (females: 588, 51.3%; males: 559, 48.7%).

A percentage of 15.4 of examined dogs (177/1147) showed an IFAT titer ≥ 1:80 and thus were found to be seropositive to CanL, while 7.4% (85/1147) were classified as exposed, showing fluorescent promastigotes at a maximum IFAT dilution titer of 1:40.

The specific anti-*L. infantum* IgG antibody titers ranged from 1:40 to 1:10,240. More specifically, 11.4% (131/1147) showed a titer ≥ 1:160, and 8.6% (99/1147) of examined dogs showed an IFAT titer ≥ 1:320.

Based on the physical examination, clinical signs consistent with CanL were described in 44.1% (78/177) of seropositive animals (titer ≥ 1:80).

General clinical signs were described in 29.4% (54/177) of seropositive dogs, including poor body condition (20.9%; 37/177) and generalized lymphadenomegaly (14.7%; 26/177). Most commonly found clinical signs were cutaneous and mucocutaneous lesions (37.9%; 67/177), exfoliative dermatitis (24.3%; 43/177), ulcerative dermatitis (13.6%; 24/177), onychogryphosis (18.1%; 32/177), truffle hyperkeratosis (5.1%; 9/177), and ocular lesions (5.1%; 9/177). Clinical signs consistent with CanL were also found in 19.6% of non-seropositive dogs.

CanL antibodies were found significantly more often in males (18.1%; 101/559) than in females (12.9%; 76/588) (*P* = 0.02). Furthermore, the number of seropositive animals seems to increase with age and is supported by a statistically significant correlation between infection and age (< 1 to 20 years) (*P* = 3e-14). No correlation (*P* > 0.05) was observed between infection and animal size, although a slight increase in prevalence seems to be in accordance to dogs size (small size: 12.9%; medium size: 14.7%; big size: 18.6%). The same can be said between infection prevalence in short-haired dogs (14.8%) compared to long-haired dogs (16.2%) (*P* > 0.05).

Prevalence rates found in purebred dogs (29.6%) were considerably higher than those found in crossbred dogs (12.9%) (*P* = 0.007). Lastly, significantly more owned dogs were found to be positive (27.2%) compared to kennel dogs (10.6%) (*P* = 0.005). Likewise, exposure was significantly higher in owned dogs (11.6%) than kennel dogs (5.7%) (*P* = 0.0004).

### Kennel dogs

Among kennel dogs, 10.6% (86/812) tested positive on IFAT, and 5.7% (46/812) of animals were classified as solely exposed to *L. infantum*. Furthermore, among seropositive dogs, 6.9% (56/812) showed a titer ≥ 1:160, and 4.7% (38/812) showed an IFAT titer ≥ 1:320.

Bivariate regression analysis detected sex, breed, and size of the animals as variables with a statistically significant relationship to infection. Indeed, male dogs have an increased risk of infection of 1.11 (CI = 1.04–1.18; *P* = 0.031) while crossbred dogs have a decreased risk of 0.74 (CI = 0.62–0.89; *P* = 0.002). Regarding body size, each increasing size increment (small-medium-large) increases the risk of infection by 1.16 (CI = 1.03–1.32; *P* = 0.04). See Table 1 for details on bivariate analysis performed on kennel dogs. The final model obtained from multivariate analysis (through backward selection within the GLMM) identified sex and breed as definitive predictors for infection in kennel dogs. Following, according to the model, risk of infection rises for male dogs with OR = 1.06 (CI = 1.01–1.10; *P* = 0.01) and decreases for crossbred dogs with OR = 0.77 (CI = 0.68–0.88; *P* = 7.7e-5) (see Table 2).

**Table 1:** Kennel dogs: results of bivariate analysis for infected dogs with each variable expressed as odds ratios (OR)

Variables	Analyzed dogs (#)	Infected dogs	Odds ratio (97.5% CI)	*P* value
Sex			1.11 (1.04–1.18)	0.031*
Male	391	53 (13.6%)		
Female	421	33 (7.8%)		
Age (years)			0.95 (0.81–1.11)	0.630
< 1	20	0		
1	65	3 (4.6%)		
2	81	9 (11.1%)		
3	75	9 (12%)		
4	48	6 (12.5%)		
5	110	14 (12.7%)		
6	68	9 (13.2%)		
7	52	9 (17.3%)		
8	47	8 (17%)		
9	32	2 (6.25%)		
10	76	5 (6.6%)		
11	29	4 (13.8%)		
12	40	3 (7.5%)		
13	25	0		
14	17	4 (23.5%)		
15	17	0		
16	6	0		
17	2	0		
18	2	0		
Breed			0.74 (0.62 - 0.89)	0.002*
Purebred	21	8 (38.1%)		
Crossbred	791	78 (9.9%)		
Size			1.16 (1.03 - 1.32)	0.040*
Small	136	10 (7.4%)		
Medium	451	41 (9.1%)		
Large	225	35 (15.6%)		
Hair Leght			0.96 (0.91 - 1.00)	0.351
Long hair	364	35 (9.6%)		
Short hair	448	51 (11.4%)		

*Statistically significant variables with *P* < 0.05

**Table 2:** Results of multivariate analysis for infected kennel and owned dogs. Odds ratios are shown only for statistically significant variables representing useful predictors.

Variables	Kennel dogs		Owned dogs	
	Odds ratio (97.5% CI)	*P* value	Odds ratio (97.5% CI)	*P* value
Sex	1.06 (1.01–1.10)	0.010*	-	-
Age	-	-	1.02 (1.00–1.03)	0.008*
Breed	0.77 (0.68–0.88)	7.7e-5	-	-
Dog’s hair	-	-	0.90 (0.81–0.99)	0.027*
Type of habitat	-	-	1.11 (C1.01–1.22)	0.035*

*Statistically significant variables with *P* < 0.05

Concerning the risk of exposed dogs to becoming infected, the final model detected sex and breed as useful predictors in this case as well. According to the model, male (OR = 1.14; CI = 1.04–1.24; *P* = 0.004) and crossbred dogs (OR = 0.48; CI = 0.37–0.62; *P* = 5e-8) have an increased and decreased risk of becoming infected, respectively (see Table 3).

**Table 3:** Results of multivariate analysis aimed at finding variables involved in the chance of exposed dogs in becoming infected. Odds ratios are shown only for statistically significant predictors

Variables	Kennel dogs		Owned dogs	
	Odds ratio (97.5% CI)	*P* value	Odds ratio (97.5% CI)	*P* value
Sex	1.14 (1.04–1.24)	0.004*	-	-
Age	-	-	1.03 (1.01–1.06)	0.008*
Breed	0.48 (0.37–0.62)	5e-08	-	-
Dog’s hair	-	-	0.80 (0.66–0.98)	0.027*
Type of habitat	-	-	1.23 (1.02–1.49)	0.035*

*Statistically significant variables with *P* < 0.05

**Fig. 1:**
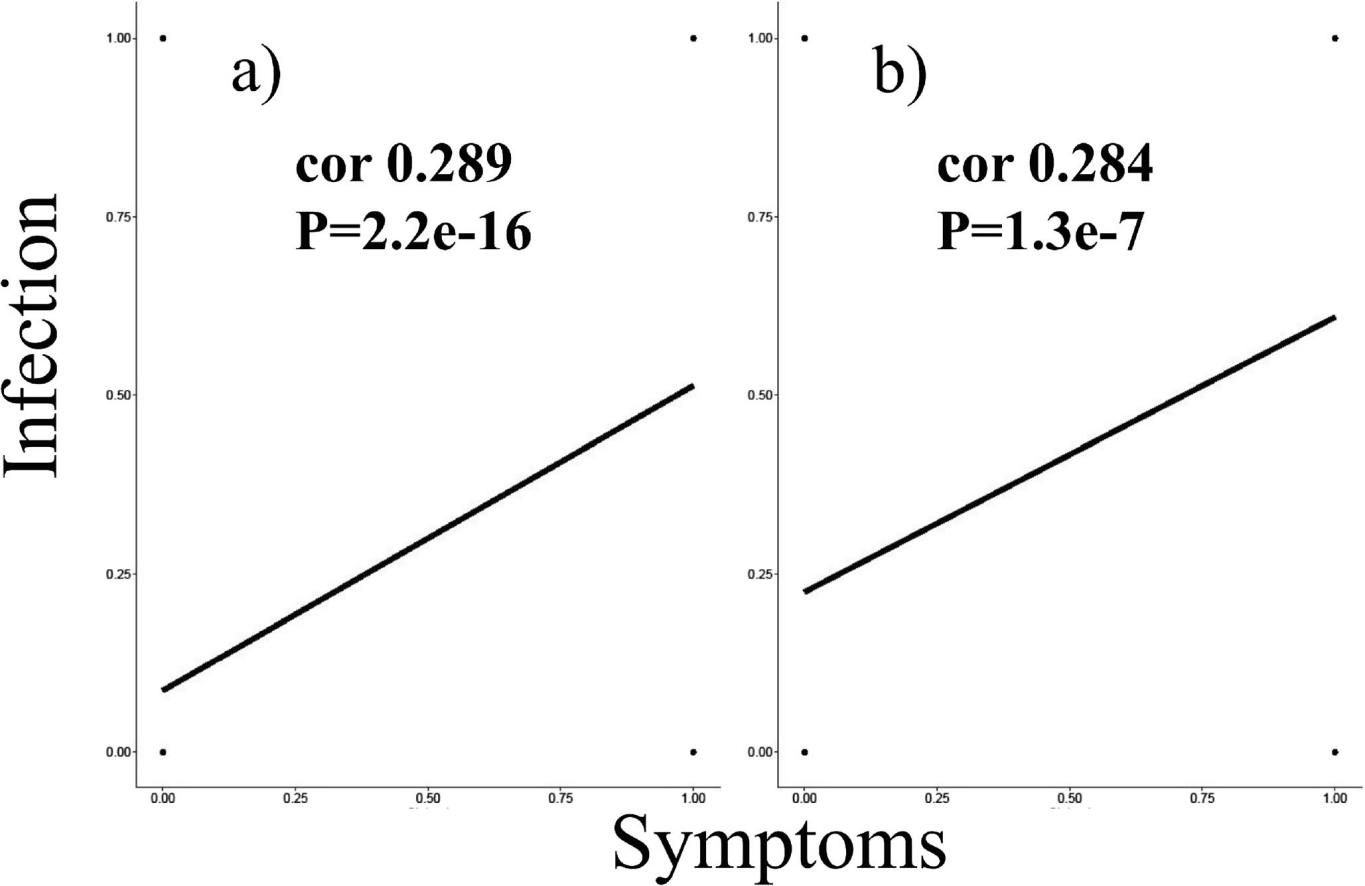
Correlation graphs. Figure of the correlation between the condition of infection and the occurrence of clinical signs in kennel and owned dogs (a, b)

Correlation analysis revealed a moderate positive association between the occurrence of clinical signs and infection (*r =* 0.289; *P* = 2.2e-16) (see Fig. 1a).

All the kennels involved in this study are located in rural areas and all the dogs housed remained outdoors during both day and night; for this reason, it was not possible to compare these variables for kennel dogs.

Veterinary health directors of the different shelters reported the use of preventives control measures against vectors in all sampled dogs and, in most cases, spot-on formulations based on pyrethroids were used.

### Owned dogs

Among owned dogs, 27.2% (91/335) of examined animals tested positive on IFAT, and 11.6% (39/335) of animals were exposed to *L. infantum*.

Regarding titration, 22.4% (75/335) of samples had a titer ≥ 1:160, and 18.2% (61/335) had an IFAT titer ≥ 1:320.

Bivariate regression analysis detected age, hair length, and type of habitat as variables with statistical significance relationships to infection. Increasing age showed to cause an increasing risk of infection with each subsequent year of life with OR = 1.45 (CI = 1.19–1.77; *P* = 0.005). Having long hair decreases the risk of infection with OR = 0.89 (CI = 0.83–0.95; *P* = 0.029), while living in rural areas increases the risk of infection with OR = 1.14 (1.06–1.23; *P* = 0.020). See Table 4 for details on bivariate analysis performed on owned dogs. In owned dogs, the final model derived from the GLMMs detected age, hair length, and type of habitat as definitive predictors for infection. According to the model, the risk of infection rises with each year of life with OR = 1.02 (CI = 1.00–1.03; *P* = 0.008). Dogs with long hair have a decreased risk of infection with OR = 0.90 (CI = 0.81–0.99; *P* = 0.027) while dogs living in rural habitats have an increased risk by OR = 1.11 (CI = 1.01–1.22; *P* = 0.035) (see Table 2).

**Table 4:** Owned dogs: results of bivariate analysis for infected dogs with each variable expressed in odds ratios (OR)

Variables	Analyzed dogs (#)	Infected dogs	Odds ratio (97.5% CI)	*P* value
Sex			1.03 (0.96–1.09)	0.617
Male	168	48 (28.6%)		
Female	167	43 (25.7%)		
Age (years)			1.45 (1.19–1.77)	0.005*
< 1	12	1 (8.3%)		
1	26	3 (11.5%)		
2	38	3 (7.9%)		
3	30	10 (33.3%)		
4	30	8 (26.7%)		
5	32	7 (21.9%)		
6	36	12 (33.3%)		
7	32	11 (34.4%)		
8	18	5 (27.8%)		
9	12	6 (50%)		
10	27	12 (44.4%)		
11	7	4 (57.1%)		
12	12	4 (33.3%)		
13	6	2 (33.3%)		
14	7	1 (14.3%)		
15	6	1 (16.7%)		
16	3	1 (33.3%)		
20	1	0		
Breed			0.95 (0.89–1.02)	0.361
Purebred	154	44 (28.6%)		
Crossbred	182	47 (25.8%)		
Size			1.01 (0.90–1.13)	0.897
Small	80	18 (22.5%)		
Medium	169	50 (29.6%)		
Large	86	23 (26.7%)		
Dog’s hair			0.89 (0.83–0.95)	0.029*
Long hair	136	46 (33.8%)		
Short hair	199	45 (22.6%)		
Prevention			0.97 (0.90–1.05)	0.612
Yes	212	56 (26.4%)		
No	231	35 (15.2%)		
Type of habitat			1.14 (1.06–1.23)	0.020*
Rural	146	47 (32.2%)		
Urban	189	44 (23.3%)		
Day shelter			0.88 (0.77–1.02)	0.142
Indoors	126	35 (27.8%)		
Outdoors	209	56 (26.8%)		
Night shelter			1.1 (0.97–1.25)	0.247
Indoors	172	45 (26.2%)		
Outdoors	163	46 (28.2%)		

*Statistically significant variables with *P* < 0.05

Concerning the risk of exposed dogs to becoming infected is concerned, our data indicate age, hair length, and type of habitat as useful predictors in this case as well. Indeed, the final model indicated that this risk rises with each year of life with OR = 1.03 (CI = 1.01–1.06; *P* = 0.008). Furthermore, dogs with long hair experience a decreasing risk with OR = 0.80 (CI = 0.66–0.98; *P* = 0.027), while dogs living in rural habitats showed an increasing of risk with OR = 1.23 (CI = 1.02–1.49; *P* = 0.035) (see Table 3).

Correlation analysis revealed moderate positive association between the occurrence of clinical signs and infection (*r* = 0.284; *P* = 1.3e-7) (see Fig. 1b).

## Discussion

The present study represents the first large-scale sero-epidemiological survey on leishmaniasis on Sardinia. Although the island has been considered endemic for this disease since the 1990s, no epidemiological study has ever been carried out. This survey provides for the first time a picture of the current epidemiological scenario of CanL on Sardinia, including the main clinical signs and risk factors associated.

The use of IFAT together with ELISA is established as the WHO’s reference technique for performing CanL surveillance studies and the determination of infection prevalence since the 1980s (WHO 1984, 2010; Morales-Yuste et al. 2012) and has long been considered to be the “gold standard” of serological methods by certain authors (Gradoni 2002; Maia and Campino 2008; Santoro and Vellusi 2015). This test, which uses whole body parasites as antigen, is useful not only in epidemiological studies, but also in clinic practice and treatment follow-up (Gradoni 2002; Alvar et al. 2004; Maia and Campino 2008). For these reasons, IFAT was selected as the serological technique in this study.

The overall seroprevalence found during this research (15.4%) is consistent with the 17.7% (range of 11–21%) previously reported for the continental Italy, a figure which was acquired through the processing of results reported in a historical CanL database including cross-sectional surveys, prospective surveys, laboratory records, cases from veterinary clinics, and case reports published since 1965 screened in the context of the EDEN subproject (Emerging Diseases in a changing European eNvironment) on leishmaniasis (Franco et al. 2011).

Results obtained here seem comparable to those reported in Bosnia and Herzegovina, where an overall seroprevalence of 16.7% was detected, with owned dogs (31.6%) more often infected compared to stray (15.3%) or shelter (14.6%) dogs (Colella et al. 2019). To this regard, owned dogs could be more susceptible to infection due to their more sedentary lifestyle and often being confined to restricted living spaces, allowing to easily be found, bitten, and infected by vectors. Furthermore, being kept near human residences is associated with an increased risk of Leishmania infection in dogs (Curi et al. 2014). In addition, all kennel dogs sampled were subjected to preventives control measures to vectors, while no treatment was reported in 15.2% of the owned dogs.

By comparing the overall prevalence found in this survey with those reported in other serological surveys carried out with the IFAT technique at the same cut-off, it can be noted the prevalence herein found to be higher than those reported in Northern Italy (2.1%; Maroli et al. 2008) and Spain (8.1%; Gálvez et al. 2010). On the other hand, Brianti et al. (2014) reported similar results for two different sites on Sicily (22.0% and 25.7%) and a much higher prevalence was found in owned dogs on the Aeolian Islands (34.6%; Otranto et al. 2017a, b) and on the island of Lampedusa (54.13%, Foglia Manzillo et al. 2018).

Kennel dogs in our study showed a higher prevalence (10.6%) value compared to those reported in other surveys. Seroprevalence of *L. infantum* in stray dogs in the Madrid region varied between 4.7 and 5.4% (Miró et al. 2017). A prevalence rate of 2.8% was found for the 18,806 dogs included in a large survey conducted between 2007 and 2012 among dogs in public kennels in Northern Italy (Emilia-Romagna region) (Santi et al. 2014). A more recent survey in kennel dogs in central Italy showed a 2.5% seroprevalence (Sauda et al. 2018). Although these last two studies were carried out using a cut-off IFAT titer of ≥ 1:160, their findings are still lower compared to the seroprevalence found in kennel dogs in the present survey when using the same cut-off (6.9%).

However, as pointed out by other Authors (Franco et al. 2011; Silva et al. 2018; Velez et al. 2019), caution must be taken when comparing studies with different experimental designs (the study area, the diagnostic method, and the sampling method) and different selection criteria for the target dog population as this can introduce significant variations in seroprevalence results.

Less than half of the seropositive dogs in this study (44.1%) showed clinical signs consistent with CanL. Mostly, general clinical signs and cutaneous lesions, that do not differ from those usually described, were found and were all related to chronic form of the disease characterized by a high IFAT titer (Miró et al. 2017; Foglia Manzillo et al. 2018). Most of the seropositive dogs were described as clinically healthy, without showing any clinical signs. This finding confirms the hypothesis made by various authors that, in endemic areas, clinical CanL representations constitute only a fraction of the leishmaniasis cases and that the majority of the dog population in such areas are exposed and become infected without showing any clinical evidence of disease or serum anti-*Leishmania* antibodies. Next, a more consistent number of dogs does have specific antibodies but no clinical signs, and a third and more numerous group of dogs is composed of healthy, antibody negative, PCR positive dogs (Baneth et al. 2008; Pennisi 2015). This being said, subclinical infection is not necessarily a permanent situation and factors such as immunosuppression and/or concomitant disease could break the equilibrium leading to the progression of clinical disease as has previously been observed in humans coinfected with HIV (Baneth et al. 2008; Solano-Gallego et al. 2009, 2011).

The risk factors analysis in this study, similar to other surveys, revealed a significantly higher prevalence in male dogs compared to females despite both groups being kept in the same way (Solano-Gallego et al. 2009; Belo et al. 2013). Conversely, gender-related differences in the host immune response might also play a role associated to the resistance and susceptibility to infection. The latter might be related to the immunomodulating properties of testosterone in dogs (Zivicnjak et al. 2005) as was previously shown in hamsters for New World leishmaniasis (Travi et al. 2002) and in human macrophages for *L. donovani* (Zhang et al. 2001).

In the present survey, seroprevalence increased with each year of life, and thus, as reported by most authors, age seems to be an important factor in the acquisition of CanL (Matos et al. 2006; Srivastava et al. 2011; Solano-Gallego et al. 2011; Mirò et al. 2012; Velez et al. 2019). Overall, the effect of age can be explained by an incremental risk of exposure to infected sand flies (Solano-Gallego et al. 2011; Mirò et al. 2012; Velez et al. 2019). Among the various hypotheses, Matos et al. (2006) stated that adult dogs tend to remain outside for longer periods of time which could increase their chances of coming into contact with the insect vector. Alternatively, the higher frequency of CanL positive results among older dogs could also be explained by the nature of the serological response to the disease. Since a long serological latency after infection might be true (Oliva et al. 2006) and animals can remain seropositive for long periods of time, increasing seroprevalence with age seems logical (Srivastava et al. 2011). Moreover, the statistical analysis of the prevalence rates of each age class of kennel dogs in our survey showed an initial increase in seroprevalence (in the first three classes) followed by a decrease in the last group of dogs (> 6 years). These findings are in agreement with other studies and could be related to an increase in mortality in older animals as supposed by Zivicnjak et al. (2005).

An effect of the animals size on infection rates was only found for kennel dogs where increasing size (small-medium-large) increased the risk of infection. Conceivably, this observation could be linked to a target size effect or differences in heat and CO_2_ irradiation between small and large sized dogs, facilitating the finding of larger hosts by the vectors, as supposed by Curi et al. (2014).

Having long hair decreases the risk of infection in owned dogs as was also found in the majority of related studies (Coura-Vital et al. 2011; Belo et al. 2013). Presumably, having a thicker haircoat inhibits the ability of vectors to feed on the blood of hosts, preventing transmission. Additionally, longer hair would cause lower CO_2_ emission and heat irradiation from the host’s body, making these dogs a less obvious target for sand flies (Coura-Vital et al. 2011; Belo et al. 2013).

Purebred dogs were found to be more often infected compared to crossbreed dogs and this is in agreement with most of the available literature (Belo et al. 2013). Some dog breeds such as the Boxer, Cocker Spaniel, Rottweiler, and German Shepherd appear to be more susceptible to the development of the disease, but these results do not allow definitive conclusions to be drawn concerning the susceptibility of individual breeds to leishmaniasis (Solano-Gallego et al. 2011). In this survey, habitat was identified as a risk factor, with dogs from rural areas being more exposed to infection. This finding corresponds to those reported in similar studies and could be due to dogs being in close proximity to non-domestic animals and to disease vectors, given that transmission can also occur in the wild (Oliveira et al. 2016). However, other studies (Queiroz et al. 2009) reported higher prevalence among dogs living in urban environments and others did not detect any effect of type of habitat at all (Velez et al. 2019). Moreover, urban and peri-urban areas offer, with their many gardens and abundance of vertebrate hosts, the ideal microclimate for the proliferation of vectors and are increasingly described as the most suitable ecosystems for the spread of CanL (Alvar et al. 2004; Ballart et al. 2013).

Regarding housing, no difference was found between dogs that slept indoor and dogs who slept in the garden as well as between dogs that had free outside access and dogs that spent the days indoor and only went out for short walks. These factors are controversial, with some authors suggesting it is not relevant whether animals are kept mainly indoors or outdoors (Zivicnjak et al. 2005), and others who claim that serological positivity to *Leishmania* sp. is significantly associated with an outdoor lifestyle (Gálvez et al. 2010; Coura-Vital et al. 2011; Belo et al. 2013; Oliveira et al. 2016). Moreover, sedentary animals and those that remain in restricted spaces seemed to have a greater risk of infection as they represent easier targets for the sand flies (Curi et al. 2014).

No difference was observed between the prevalence found in dog regularly subjected to preventive control measures against arthropods and dogs where owners did not apply such measures. Similar results have also been reported in other studies (Foglia Manzillo et al. 2018). Although the effectiveness of some formulations in the prevention of CanL transmission is well documented, improper use of these treatments, such as non-application to all dogs or non-maintenance during the whole season of transmission, may compromise the protective effect of these products (Brianti et al. 2014).

The present survey confirmed the endemicity of CanL in Sardinia, and considering that two proven *L. infantum* vectors (i.e., *P. perfiliewi* and *P. perniciosus*) have been recently confirmed on the island, this may indicate a risk factor not only for dogs but also for humans (Carta et al. 2020).

In 2018, over three million tourists visited Sardinia and more than one million visitors traveled from areas within northern Europe where leishmaniasis is not endemic (e.g., Germany, France, Switzerland, Netherlands, and the UK) (Carta et al. 2020). This high mobility increases the possibility of transmission of *L. infantum* overseas especially because these tourists are often accompanied by their pets (dogs), which might transport the disease back North after getting infected when visiting endemic areas (such as Sardinia) (Carta et al. 2020). In a recent German study carried out on dogs which had recently traveled to the Mediterranean, most of which had accompanied their owners to Italy, 5.0% were found to be infected with *L. infantum* (Schäfer et al. 2019); in addition, more than 700 imported CanL cases have been reported in traditionally non-endemic countries in Europe in the last few years (Maia and Cardoso 2015). Although the overall risk of being infected with *L. infantum* is probably low since pets traveling with their owners usually do not stay at their holiday destinations for prolonged periods of time, owners should be advised to use preventive measures for the protection of their dogs when traveling to endemic areas like Sardinia in any case. Additionally, because of *L. infantum*’s zoonotic potential, the application of such prophylactic methods is not only important for animal health but also for human and public health in Europe (Schäfer et al. 2019; Maia and Cardoso 2015).

## Conclusions

In conclusion, the results obtained within this survey confirm the endemicity of CanL on the island of Sardinia as previously reported for analogous areas in the Mediterranean. Additionally, a deepened understanding of the risk factors associated with canine infection was generated although some enigmatic or difficult to interpret factors remain. Prevalence rates found here underline the need for the application of acute control measures which very much coincide with a “One Health” approach against leishmaniasis. In particular, preventive measures against phlebotomine sand fly bites have proven central in fighting this disease and should be fulfilled using appropriate products for the complete duration of the transmission season.
